# Evaluation of the Performance of a Multiplexed Serological Assay in the Detection of SARS-CoV-2 Infections in a Predominantly Vaccinated Population

**DOI:** 10.1128/spectrum.01454-21

**Published:** 2022-02-23

**Authors:** Michael Asamoah-Boaheng, David M. Goldfarb, Vilte Barakauskas, Tracy L. Kirkham, Paul A. Demers, Mohammad Ehsanul Karim, Pascal M. Lavoie, Ana Citlali Marquez, Agatha N. Jassem, Sandra Jenneson, Christopher MacDonald, Brian Grunau

**Affiliations:** a Faculty of Medicine, Clinical Epidemiology, Memorial University of Newfoundland, Newfoundland and Labrador, Canada; b Department of Emergency Medicine, University of British Columbia and St. Paul’s Hospital, Vancouver, British Columbia, Canada; c Department of Pathology and Laboratory Medicine, University of British Columbia, Vancouver, British Columbia, Canada; d Dalla Lana School of Public Health, University of Toronto, Toronto, Ontario, Canada; e Occupational Cancer Research Centre, Ontario Health, Toronto, Ontario, Canada; f School of Population and Public Health, University of British Columbia, Vancouver, British Columbia, Canada; g Centre for Health Evaluation & Outcome Sciences, University of British Columbia, Vancouver, British Columbia, Canada; h Department of Pediatrics, University of British Columbia, Vancouver, British Columbia, Canada; i British Columbia Emergency Health Services, Vancouver, British Columbia, Canada; Quest Diagnostics Nichols Institute

**Keywords:** SARS-CoV-2, COVID-19, nucleocapsid, test performance, vaccination

## Abstract

SARS-CoV-2 seroprevalence studies may be complicated by vaccination efforts. It is important to characterize the ability of serology methods to correctly distinguish prior infection from postvaccination seroreactivity. We report the performance of the Meso Scale Discovery (MSD) V-PLEX COVID-19 Coronavirus Panel 2 IgG assay. Using serum samples from a prospective cohort of paramedics, we calculated the performance of the V-PLEX nucleocapsid (“N”) assay to classify prior SARS-CoV-2 infections, defined as a (i) history of a positive SARS-CoV-2 PCR test or (ii) positive serology results using the Roche Elecsys total nucleocapsid anti-SARS-Cov-2 assay. We calculated sensitivity and specificity at the optimal threshold (defined by the highest Youden index). We compared subgroups based on vaccination status, and between models that excluded prior infections 3 to 12 months before sample collection. Of 1119 participants, 914 (81.7%) were vaccinated and 60 (5.4%) had evidence of a preceding SARS-CoV-2 infection. Overall and within vaccinated and unvaccinated subgroups, the optimal thresholds were 828 AU/mL, 827 AU/mL, and 1324 AU/mL; with sensitivities of 0.95 (95% CI: 0.94 to 0.96), 0.95 (0.94 to 0.96), 0.94 (0.92 to 0.96) and specificities of 0.88 (0.86 to 0.90), 0.87 (0.85 to 0.89), and 0.94 (0.89 to 0.98), respectively. N-assay specificity was significantly better in unvaccinated (versus vaccinated) individuals (*P* = 0.005). Overall optimal thresholds based on the AUC values were higher for samples from unvaccinated participants, especially when examining infections within the preceding 9 months (5855 versus 1704 AU/mL). Overall, V-PLEX nucleocapsid assay cutoff values were higher among unvaccinated individuals. Specificity was also significantly higher among unvaccinated individuals. Different thresholds were required to achieve optimal test performance, especially for detecting SARS-CoV-2 infections within the preceding 9 months.

**IMPORTANCE** Among a cohort of adult paramedics in Canada, we investigated the performance of nucleocapsid (N) antibody detection (measured with a V-PLEX assay) to identify previous COVID-19 infections and compared differences among vaccinated and unvaccinated. Our data indicate that vaccinated and unvaccinated groups require different thresholds to achieve optimal test performance, especially for detecting COVID-19 within the preceding 9 months. Overall, specificity was significantly higher among unvaccinated, compared to vaccinated individuals.

## OBSERVATION

The virus that causes coronavirus disease 2019 (COVID-19), severe acute respiratory syndrome-related coronavirus-2 (SARS-CoV-2), was identified as the cause of an international pandemic by the World Health Organization on March 20, 2020 (1). By November 2021, SARS-CoV-2 had caused over 5 million deaths (2).

Serological assays to measure SARS-CoV-2 antibodies have been developed, which have been used to estimate seroprevalence for epidemiological descriptions or comparative studies (3). These tests play a key role in quantifying the cumulative incidence of COVID-19 given that not all cases undergo PCR testing to identify symptomatic or asymptomatic infections (4). Information from these assays may become increasingly vital as more and more individuals are vaccinated, leading to milder SARS-CoV-2 infections (5), which may elicit less enthusiasm for testing and, thus, less case identification. However, identifying COVID-19 in the postvaccine era among vaccinated and unvaccinated individuals alike is of critical importance to monitor vaccine effectiveness (especially pertaining to variants of concern) for COVID-19 surveillance and to better understand dynamics of potentially waning immunity in the postvaccinated population.

Knowledge of serological test performance is imperative to determine its utility and correctly estimate disease prevalence. However, antibody response and test performance may be significantly altered among vaccinated individuals and may be affected with the interval since the SARS-CoV-2 infection (6). The V-PLEX COVID-19 coronavirus panel 2 IgG assay measures four SARS-CoV-2 antigens (spike, receptor-binding domain [RBD] of S1, N-terminal domain of the spike protein [NTD], and the nucleocapsid [N] protein). V-PLEX spike antibody concentrations have demonstrated high sensitivity and specificity for identifying preceding COVID-19 in nonvaccinated individuals with improved performance when sample collection occurred later after symptom onset (the longest interval analyzed was >21 days) (7). Because many individuals are now vaccinated with spike-based vaccines (8), spike antigen targets are largely unusable for identifying prior infections. While nucleocapsid antibody measurements may have utility for this purpose, their performance among vaccinated individuals is unclear. Further, although previous evaluations showed improved sensitivity from the time of symptom onset, intervals were relatively short (i.e., less than 3 months), and, thus, the longevity of the antibody response requires further investigation.

We sought to assess the performance of the V-PLEX N assay in identifying prior SARS-CoV-2 infections, using a large cohort of prospectively enrolled subjects, many of whom were vaccinated and some with documented COVID-19 infections. We compared the performance of the V-PLEX N assay among vaccinated and nonvaccinated cohorts and with increasing intervals from the SARS-CoV-2 infection date.

Our study employed data from the COVID-19 Occupational Risks, Seroprevalence, and Immunity among Paramedics (CORSIP) in Canada study (commenced January 2021). The first COVID-19 case in Canada was documented on January 15, 2020 (9). The CORSIP study is an observational prospective cohort design that enrolled adult (age ≥19) paramedics working in western Canada or Ontario during the COVID-19 pandemic. The overarching goal of the CORSIP study was to investigate occupational risks and seroprevalence of SARS-CoV-2 immune measures among paramedics. Participants were requested to provide a blood sample upon study enrollment, complete a questionnaire (including demographic characteristics, vaccination status, occupational risk factors), and document dates and results of all SARS-CoV-2 PCR tests and vaccinations.

With regard to the study participants, we included CORSIP participants in this analysis who had provided blood samples between January 8, 2020, and June 9, 2021.

Each serum sample was tested using (i) the Meso Scale Discovery V-PLEX COVID-19 Coronavirus Panel 2 IgG assay (Meso Scale Diagnostics, MD, USA), to evaluate the detection of IgG antibodies binding to the N SARS-CoV-2 antigen and (ii) the Roche Elecsys Anti-SARS-Cov-2 N assay (Roche Diagnostics Corp., IN, USA), which is a Food and Drug Administration-approved assay to identify individuals with prior SARS-Cov-2 infections that had demonstrated high sensitivity and specificity (10) (Text S1).

We assessed the study outcomes by utilizing two different reference standards for evaluation of the V-PLEX N assay to identify prior COVID-19. In our primary analysis, we defined prior COVID-19 as individuals with a positive SARS-CoV-2 PCR test or a reactive Elecsys N assay result. We used these measures in combination as COVID-19 may have been missed by PCR testing due to asymptomatic infections or incomplete PCR testing for symptomatic infections. We also performed secondary analyses defining COVID-19 using only PCR results given possible misclassification of the Elecys N assay.

We used SPSS v 27 (IBM; NY, USA) and R version 3.6.2 for statistical analyses. We described the characteristics of the study population and categorized them by vaccination status. We compared group characteristics with the Wilcoxon sign-rank test (to compare medians of continuous covariates) and Chi-square test (to compare categorical variables).

In our primary analysis, we calculated the performance of the V-PLEX N assay in classifying subjects as having a preceding SARS-CoV-2 infection, using PCR and Elecsys N for the reference standard. We calculated the sensitivity and specificity (with 95% confidence interval) at the optimal threshold (defined as the highest Youden index) and estimated the area under the curve (AUC) using the receiver operating characteristic (ROC). We repeated the analysis using only PCR results for the reference standard.

We performed additional secondary analyses. First, we calculated the lowest threshold values for which sensitivity and specificity were ≥0.95. Second, we calculated the sensitivity and specificity using the V-PLEX manufacturer’s recommended threshold of 5000 AU/liter. Third, to compare test performance between vaccinated and unvaccinated subjects, we compared the AUC, sensitivity, and specificity using “independent group area difference” and “Delong’s test for correlated ROC curves” tests (11). Fourth, as it is possible that V-PLEX N testing may not be able to detect remote SARS-Cov2 infections, we performed a series of sensitivity analyses in which we only considered positive PCR tests in the preceding 3 to 6, 9, or 12-month periods before blood sampling, and compared test performance (to the 12-month reference). As serology testing is not able to distinguish the time of the COVID-19 infection, we used only PCR test results as the reference standard for this secondary analysis.

Of 1,119 participants who provided blood samples, 914 (82%) were vaccinated (770 BNT162b2 [Pfizer]; 144 mRNA-1273 [Moderna]). Blood samples were provided between January 8, 2020 and June 9, 2021. The median age was 38 years (IQR: 31 to 47 years; Table 1).

**TABLE 1:** Participant characteristics, overall and stratified by vaccination status

Study characteristics	Overall	Vaccination status		*P* value^*a*^
	N^*b*^ = 1119	Vaccinated N = 914	Unvaccinated N = 205	
Age & Sex				
Age (yrs)^*c*^, median (IQR)	38 (31-47)	38 (31-47)	37 (30-44)	0.248
Female sex at birth, n (%)	502 (45)	407 (45)	95 (46)	0.155
Male sex at birth, n (%)	613 (55)	505 (55)	108 (53)	
Ethnicity/Race, n (%)				
White	981 (88)	801 (88)	180 (88)	0.697
Asian	123 (11)	102 (11)	21 (10)	
Others	15 (1)	11 (1)	4 (2)	
Educational level, n (%)				
Non-university certificate	729 (65)	580 (64)	149 (73)	0.004
University Bachelor’s degree	348 (31)	296 (32)	52 (25)	
University Graduate degree	34 (3)	33 (4)	1 (1)	
Smoking history, n (%)				
Cigarette use	52 (5)	45 (5)	7 (3)	0.085
E-cigarette use	47 (4)	35 (4)	12 (6)	0.083
Past Medical History, n (%)				
Hypertension	95 (9)	83 (9)	12 (6)	0.215
Diabetes	23 (2)	19 (2)	4 (2)	0.991
Asthma	157 (14)	135 (15)	22 (11)	0.285
Chronic lung disease	6 (1)	6 (1)		
Chronic Heart Disease	9 (1)	8 (1)	1 (1)	0.837
Liver disease	11 (1)	11 (1)		
Malignancy	24 (2)	19 (2)	5 (2)	0.741
Immune suppressed	27 (2)	21 (2)	6 (3)	0.901
Vaccine type				
Pfizer, n (%)	770 (69)	766 (84)		
Modena, n (%)	145 (13)	145 (16)		
Serology and PCR Testing Results				
SARS-CoV-2 PCR test positive, n (%)	38 (3)	30 (3)	8 (4)	0.658
Elecsys N + test, n (%)	56 (5)	40 (4)	16 (8)	
Elecsys N + and PCR−, n (%)	22 (2)	13 (1)	9 (4)	0.006
COVID-19 infection (PCR+ or Elecsys N+)	60 (5)	43 (5)	17 (8)	0.039
1^st^ vaccine-to-BS interval, median (IQR)	58 (37-93)	59 (37-93)	NA	
PCR+ to BS intervals, median (IQR)	118 (71-177)	118 (79-171)	120 (37-349)	0.753
V-PLEX Nucleocapsid, gM (gSD)	217 (5)	220 (5)	203 (6)	0.397
V-PLEX Nucleocapsid, Median (IQR)	172 (76-467)	177 (76-477)	148 (72-394)	0.247

a Significance tests were performed using a Wilcoxon sign-rank test (compare medians of continuous covariates) or Chi-square test (compared categorical variables).

b N, number; gM, geometric mean; gSD, geometric standard deviation; IQR, interquartile range; N, nucleocapsid; +, positive; −, negative; BS, blood sample.

c Age range: 20 to 83 years.

Sixty (5.4%) participants had evidence of previous COVID-19 infections on the date of blood sampling with 38 identified using PCR tests and 22 additional cases identified with reactive Elecsys N serology. Among cases with a positive PCR result, 34/38 (89%) also had reactive Elecsys N serology, where 27/30 were vaccinated and 7/8 were unvaccinated. Among the vaccinated cases with positive PCR results postvaccination, 2/2 (100%) had reactive Elecsys N serology. Of those with positive PCR tests, the PCR-to-blood sampling interval was a median of 119 days (IQR: 179 to 77 days; range 24 to 410 days). Specifically, for the 4 cases with unreactive Elecsys serology, the intervals were 71, 120, 171, and 366 days. Median V-PLEX N values overall and among vaccinated and nonvaccinated cases was 171.5 AU/mL (IQR: 75.9 to 467.4), 176.9 AU/mL (IQR: 76.5 to 477.0), and 148.4 AU/mL (IQR: 71.8 to 393.9), respectively.

Using the reference standard of PCR and Elecsys N serology, Table 2 and Fig. S1 show test characteristics of the V-PLEX N assay overall and within vaccinated and unvaccinated subgroups (additional ROC curves in Text S1), with the optimal threshold defined by the highest Youden Index. Overall, the optimal threshold was 828 AU/mL with a sensitivity and specificity of 0.95 (95% CI: 0.94 to 0.96) and 0.88 (0.86 to 0.90), respectively. Using the reference standard of PCR only, Table 2 shows test characteristics of the V-PLEX assay overall and within vaccinated and unvaccinated subgroups with the optimal threshold defined by the highest Youden Index.

**TABLE 2:** Diagnostic test performance of V-PLEX nucleocapsid assay in detecting preceding COVID-19 infections, using a reference standard of PCR and Roche nucleocapsid serology, among 1119 participants (both vaccinated and unvaccinated)

Analysis group^*a*^	COVID-19+	Optimal N threshold^*b*^	Sensitivity (95% CI)	Specificity (95% CI)	Youden index	AUC^*c*^ (95% CI)
Overall (*n* = 1119)	60 (5.36%)	828	0.95 (0.94, 0.96)	0.88 (0.86, 0.90)	0.83	0.96 (0.94, 0.99)
Vaccinated (*n* = 914)	43 (4.70%)	827	0.95 (0.94, 0.96)	0.87 (0.85, 0.89)	0.83	0.97 (0.94, 0.99)
Unvaccinated (*n* = 205)	17 (8.29%)	1324	0.94 (0.92, 0.96)	0.94 (0.89, 0.98)	0.87	0.95 (0.90, 1.00)

a Includes 914 vaccinated and 205 unvaccinated participants.

b Determined by the highest Youden Index.

c AUC, area under the curve; N, nucleocapsid; CI, confidence interval.

Table S1 shows test characteristics of the V-PLEX assay overall and within vaccinated and unvaccinated subgroups with the threshold defined by the manufacturer (5000 AU/mL).

Comparing V-PLEX N test performance within vaccinated to unvaccinated samples, there was a significant difference in specificity (Z = 2.816, P = 0.005) but not sensitivity (Z = 0.584, P = 0.562). The AUC was not significantly different between vaccinated and unvaccinated models (Delong’s test for two ROC curves; Z = 0.352; AUC difference = 0.02; P = 0.725).

Table 3 shows V-PLEX N test characteristics examining variable lengths of observation preceding the blood sampling. Compared to 12 months of preceding observation, the optimal threshold for unvaccinated samples increased from 1,460 to 5,855 AU/mL for the 9-month group, whereas it remained stable at 1,704 AU/mL for vaccinated samples. We did not detect a difference in AUC based on observation period (12-month reference) for 9 months (P = 1.000), 6 months (P = 0.999), or 3 months (P = 0.999).

**TABLE 3:** Diagnostic test performance of the V-PLEX nucleocapsid assay (optimal threshold defined by the Youden Index) using a reference standard of PCR test results

Analysis group^*a*^	PCR positive^*b*^	Optimal N threshold^*c*^	Sensitivity (95% CI)	Specificity (95% CI)	Youden index	AUC^*d*^ (95% CI)
Overall						
Eligible (*n* = 1119)	38 (3.40%)	1493	0.89 (0.88, 0.90)	0.91 (0.89, 0.93)	0.81	0.95 (0.91, 0.98)
Vaccinated (*n* = 914)	30 (3.28%)	1704	0.90 (0.89, 0.91)	0.92 (0.90, 0.94)	0.82	0.96 (0.93, 0.99)
Unvaccinated (*n* = 205)	8 (3.90%)	1460	0.86 (0.83, 0.89)	0.91 (0.87, 0.95)	0.77	0.89 (0.77, 1.00)
12-mo observation period preceding serology testing						
Eligible (*n* = 1119)	37 (3.30%)	1493	0.92 (0.91, 0.93)	0.91 (0.89, 0.93)	0.83	0.948 (0.91,0.99)
Vaccinated (*n* = 914)	29 (3.17%)	1704	0.93 (0.92, 0.94)	0.92 (0.90, 0.94)	0.86	0.962 (0.93, 0.99)
Unvaccinated (*n* = 205)	8 (3.90%)	1460	0.86 (0.89, 0.95)	0.91 (0.87, 0.95)	0.77	0.893 (0.77, 1.00)
9-mo observation period preceding serology testing						
Eligible (*n* = 1119)	33 (2.90%)	1708	0.94 (0.93, 0.95)	0.92 (0.90, 0.94)	0.86	0.961 (0.93, 0.99)
Vaccinated (914)	28 (3.10%)	1704	0.93 (0.92, 0.94)	0.92 (0.90, 0.94)	0.85	0.959 (0.93, 0.99)
Unvaccinated (*n* = 205)	5 (2.40%)	5855	1.00 (0.98, 1.02)	0.96 (0.92, 1.00)	0.96	0.977 (0.96, 0.99)
6-mo observation period preceding serology testing						
Eligible (*n* = 1119)	33 (2.90%)	1708	0.94 (0.93, 0.95)	0.92 (0.90, 0.94)	0.86	0.961 (0.93, 0.99)
Vaccinated (*n* = 914)	28 (3.10%)	1704	0.93 (0.92, 0.94)	0.92 (0.90, 0.94)	0.85	0.959 (0.93, 0.99)
Unvaccinated (*n* = 205)	5 (2.40%)	5855	1.00 (0.98, 1.02)	0.96 (0.92, 1.00)	0.96	0.977 (0.96, 0.99)
3-mo observation period preceding serology testing						
Eligible (*n* = 1119)	19 (1.70%)	1708	0.94 (0.93, 0.95)	0.91 (0.89, 0.93)	0.86	0.955 (0.91, 0.99)
Vaccinated (*n* = 914)	15 (1.60%)	1704	0.93 (0.92, 0.94)	0.91 (0.89, 0.93)	0.84	0.950 (0.90, 1.00)
Unvaccinated (*n* = 205)	4 (2.00%)	5855	1.00 (0.98, 1.02)	0.96 (0.92, 1.00)	0.96	0.976 (0.95, 1.00)

a Includes 914 vaccinated and 205 unvaccinated participants.

b PCR results only consider positive results which occurred within the period of observation for that analysis.

c Determined by the highest Youden Index.

d AUC, area under the curve; N, nucleocapsid; CI, confidence interval; MSD, meso scale discovery.

We examined data from over 1,119 paramedics enrolled in a prospective population-based study, all of whom were tested with the quantitative V-PLEX and Elecsys N protein assays. Our data indicated that different thresholds were required to achieve optimal test performance between vaccinated and unvaccinated subgroups, and we found significant differences in specificity. This observed vaccine-related reduction in specificity for a nucleocapsid-based SARS-CoV-2 serological assay has not been described previously. However, few studies have evaluated these assays in vaccinated populations. One potential explanation might be a possible “back boosting” phenomenon where vaccination results in the generation of antibody responses to previously encountered similar strains (12).

When examining test performance with decreasing infection-to-sampling intervals, we did not detect differences in overall test performance. However, it is noteworthy that, among unvaccinated individuals, the optimal threshold increased to over 5,000 AU/mL to detect COVID-19 infections within a 9-month observation period - achieving a sensitivity of 100% - while the optimal threshold for vaccinated individuals remained below 2,000 AU/mL.

While sensitivity was relatively high, overall and for all subgroups, specificity was <95%, which may be problematic to estimate seroprevalence for an uncommon disease state. However, if used as an initial screening test in an orthogonal testing strategy, this still may be useful, given the high sensitivity. Alternatively, a different threshold could be used to prioritize specificity and limit false-positives, which was the case for the manufacturer’s recommended threshold of 5000 AU/mL that achieves high specificity with low sensitivity.

A previous study, evaluating the V-PLEX N assay in unvaccinated samples with infection-to-sampling intervals of <64 days reported an AUC of 0.90 (95% CI 0.87 to 0.94) and a specificity of 93% (2). Sensitivity was highest (87%) for infection-to-sampling intervals >21 days. Our study extended these findings by demonstrating this sensitivity increased to 100% by month 3 and then decreased after 9 months.

Before vaccination, there were concerns regarding the performance of nucleocapsid-based assays due to the observed waning of seropositivity after infection when using this target (13) and, thus, spike-based assays were primarily used to identify preceding COVID-19. However, as spike-based assays no longer have utility among the vaccinated, it is particularly important to understand how nucleocapsid assay performs longitudinally in detecting infections in the broader population (8). This is important for ongoing surveillance, especially for break-through infections among vaccinated individuals, which may be significantly less severe (5) (or possibly asymptomatic) than infections among unvaccinated individuals. Thus, individuals may be less likely to pursue PCR-based testing.

Our study is subject to several limitations. First, we may have misclassified some true positive cases despite using a reference standard of PCR or Elecsys N serology testing. While our subgroup analyses examining differing preceding intervals of observation accounts for PCR testing for those with positive serology testing, we were unable to ascertain the time of infection. Our study cohort included a group of Canadian middle-aged paramedics. Results may differ among different settings and population characteristics.

In conclusion, when comparing vaccinated and unvaccinated subgroups, we found significant differences in specificity. Different thresholds were required to achieve optimal test performance, especially for detecting SARS-CoV-2 infections within the preceding 9-months. These findings have significant implications for ongoing efforts to identify break-through infections among vaccinated individuals.
