# Supplementary material for: Evaluation of the Performance of a Multiplexed Serological Assay in the Detection of SARS-CoV-2 Infections in a Predominantly Vaccinated Population
Source: Microbiol Spectr. 2022 Feb 23;10(1):e01454-21. doi: 10.1128/spectrum.01454-21 (PMC8865468; doi:10.1128/spectrum.01454-21)
Supplement: SUPPLEMENTAL FILE 1 — Supplemental material. Download SPECTRUM01454-21_Supp_1_seq4.pdf, PDF file, 0.6 MB [file spectrum01454-21_supp_1_seq4.pdf]

SUPPLEMENTAL MATERIALS

Roche Testing Procedures

All plasma samples were also tested with the Elecsys Anti-SARS-CoV-2 nucleocapsid (Roche, USA) assay, an immunoassay for the in-vitro qualitative detection of nucleocapsid antibodies (including IgG) to SARS-CoV-2.

The Roche assay was performed as per manufacturer's instructions, at a single laboratory (the Canadian Blood Services national clinical laboratory), using the same lot of reagents. Samples with an anti-SARS-CoV-2 concentration above the measuring range (250 U/ml) were diluted by the Roche analyzer with Diluent Universal at 1:10, as per manufacturer's instructions. After dilution by the analyzer, the software automatically utilized the dilution value when calculating the sample concentration, up to the maximum value of 2500 U/ml. Heat inactivation was not performed. For quality control, we used PreciControl Anti-SARS-CoV-2. These were run at least once every 24 hours. Cut-offs were determined automatically by the analyzer software based on calibrated master curves. For calibration, the method is standardized against an internal Roche standard for anti-SARS-CoV-2 provided with the assay. A pre-defined master curve is adapted to the analyzer using the kit calibration reagents. Calibration is performed once per reagent lot or following servicing of the analyzer.

MSD Testing Procedures

Nucleocapsid antibody concentrations were measured using the V-PLEX COVID-19 Coronavirus Panel 2 IgG assay (Meso Scale Discovery [MSD], Maryland, USA), reported as Arbitrary Units [AU/mL]). It utilizes a 96-well based solid-phase antigen printed plate and an electrochemiluminescent detection system measuring IgG to nine antigens, including four SARS-CoV-2 antigens (spike, receptor binding domain of S1 [RBD], N terminal domain of the spike protein [NTD], and the nucleocapsid [N] protein), as well as spike antibodies to HCoV-229E , HCoV-HKU1 , HCoV-OC43 , HCoV-NL63 , SARS-CoV-1 human coronaviruses.

The MSD assay was performed as per the manufacturer's instructions, at a single laboratory. Samples were tested at a 1:5000 dilution, with the diluent provided by the manufacturer (Diluent 100). Heat inactivation was not performed. The assay includes a standard curve based on a

reference standard that contains a pre-determined concentration of each antigen. It also includes three serological controls which contain a known concentration of IgG antibodies against the antigens of the assay. We ran the standard curve and serological controls on every plate to assess for quality control.

Supplemental Tables

	COVID-19 Reference Standard: PCR				COVID-19 Reference Standard: PCR and Elecsys Nucleocapsid			
Analysis Group[1]	COVID-19 n (%)	Sensitivity (95% CI)	Specificity (95% CI)	AUC (95% CI)	COVID-19 n (%)	Sensitivity (95% CI)	Specificity (95% CI)	AUC (95% CI)
Overall (n=1119)	38 (3.40%)	0.67 (0.66, 0.68)	0.97 (0.95, 0.99)	0.95 (0.91,0.98)	60 (5.36)	0.65 (0.64, 0.66)	0.98 (0.96, 1.00)	0.96 (0.94, 0.99)
Vaccinated (n=914)	30 (3.28%)	0.70 (0.69, 0.71)	0.97 (0.95, 0.99)	0.96 (0.93,0.99)	43 (4.70)	0.67 (0.66, 0.68)	0.98 (0.96, 1.00)	0.97 (0.94, 0.99)
Unvaccinated (n=205)	8 (3.90%)	0.57 (0.54, 0.60)	0.96 (0.92, 1.00)	0.89 (0.77.1.00)	17 (8.29)	0.59 (0.57, 0.61)	0.98 (0.94, 1.00)	0.95 (0.90, 1.00)

Table S1: Diagnostic Test Performance of V-PLEX Nucleocapsid Assay (Threshold of 5000 AU/mL, as Defined by Manufacturer), Using a reference standard of PCR test and Roche Nucleocapsid results

AUC, area under the curve; N, nucleocapsid; CI, confidence interval; PCR, polymerase chain reaction

Supplementary Figures

Supplementary Figure 1: Receiver Operating Characteristic (ROC) curves for measuringthe performance of V-PLEX N in classifying preceding SARS-Cov2 infections, overall (top), and among vaccinated (middle) and unvaccinated (bottom) cases, using a reference standard of PCR and Roche nucleocapsid serology

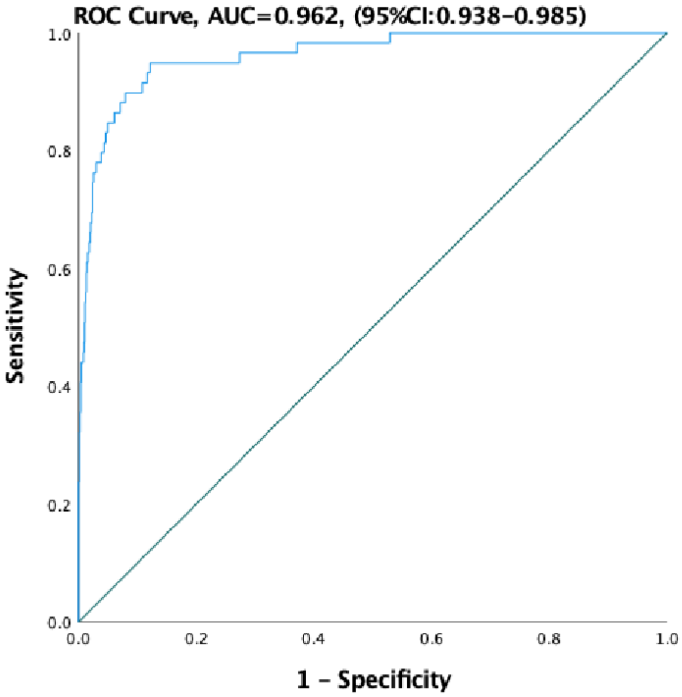

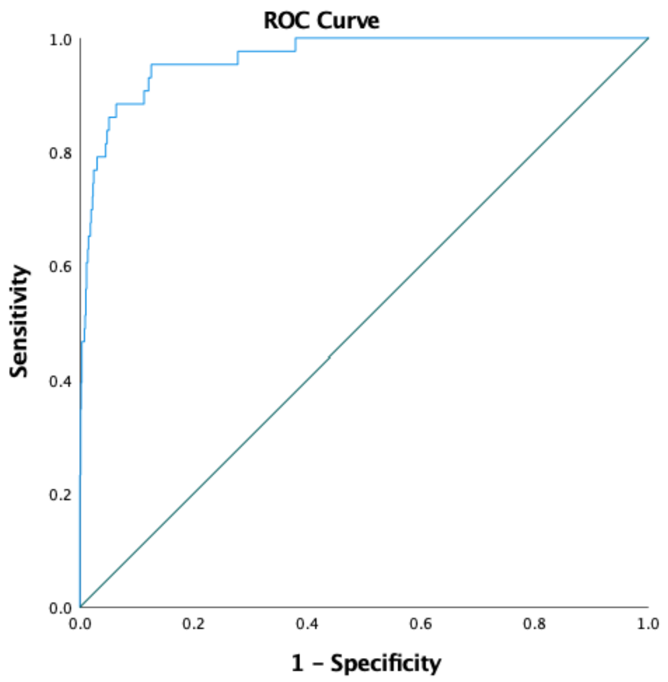
ROC Curve
Sensitivity
1.0
0.8
0.6
0.4
0.2
0.0
0.0
0.2
0.4
0.6
0.8
1.0
1 – Specificity

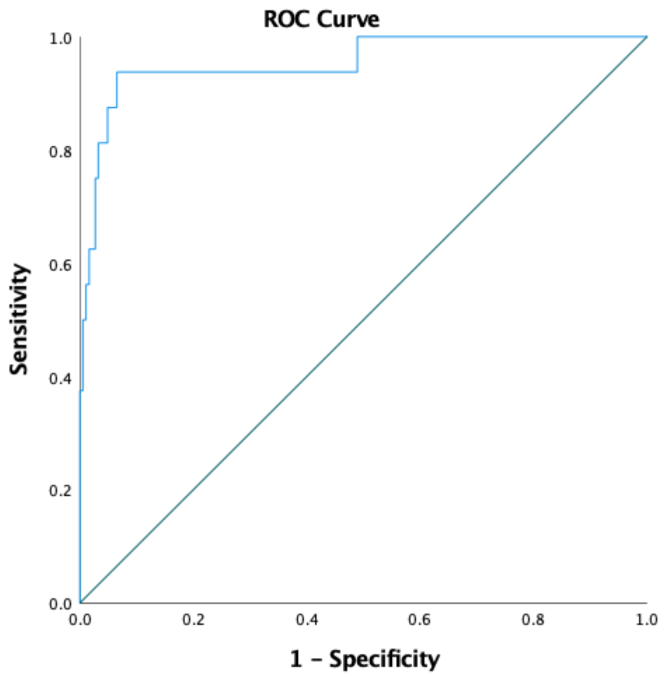
ROC Curve
Sensitivity
1.0
0.8
0.6
0.4
0.2
0.0
0.0
0.2
0.4
0.6
0.8
1.0
1 – Specificity

Supplementary Figure 2: ROC curves for measuring the performance of V-PLEX N in detecting SARS-Cov2 PCR infections with 12-months of preceding observation, overall(top), and among vaccinated (middle), and unvaccinated (bottom) cases

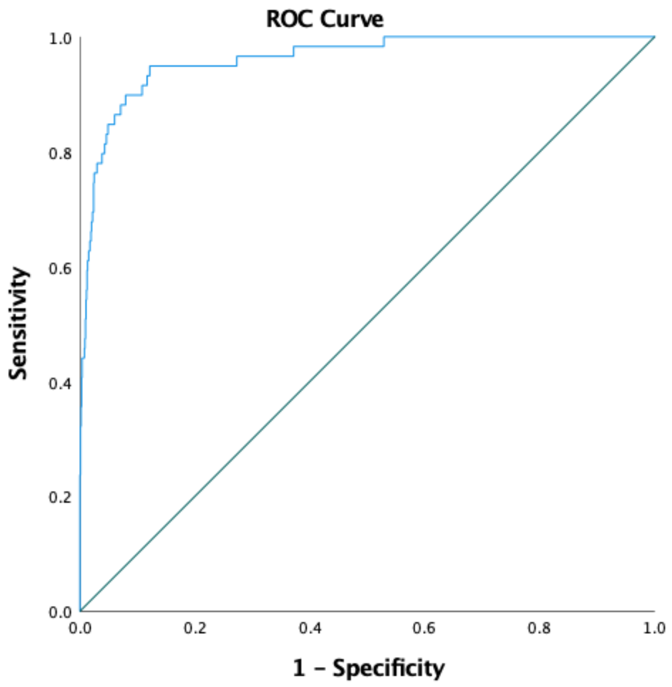

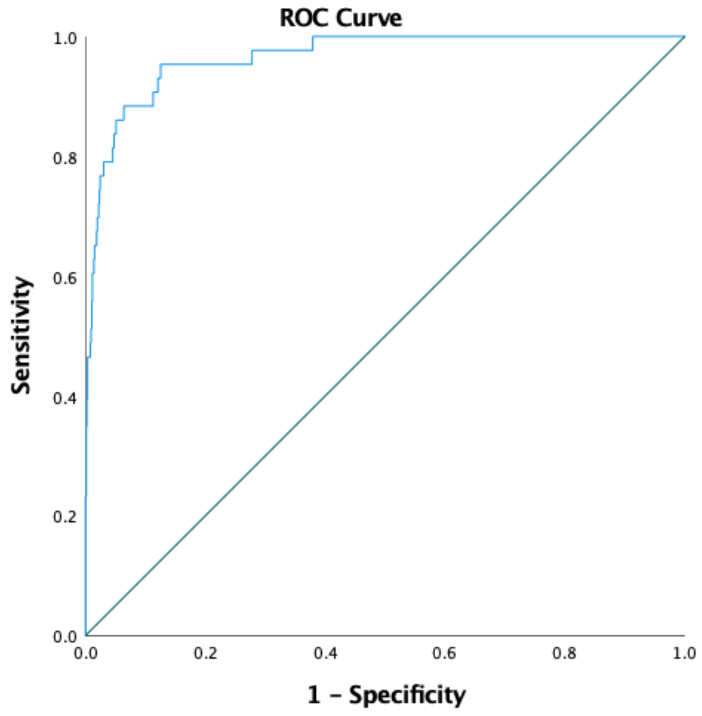

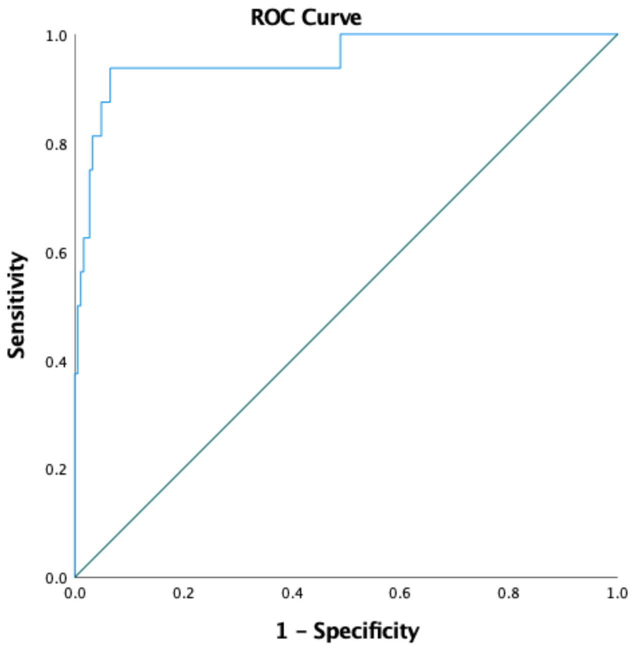
ROC Curve
Sensitivity
1.0
0.8
0.6
0.4
0.2
0.0
0.0
0.2
0.4
0.6
0.8
1.0
1 – Specificity

Supplementary Figure 3: ROC curves for measuring the performance of V-PLEX N in detecting COVID-19 infection with 9-months of preceding observation, overall (top), and among vaccinated (middle), and unvaccinated (bottom) cases

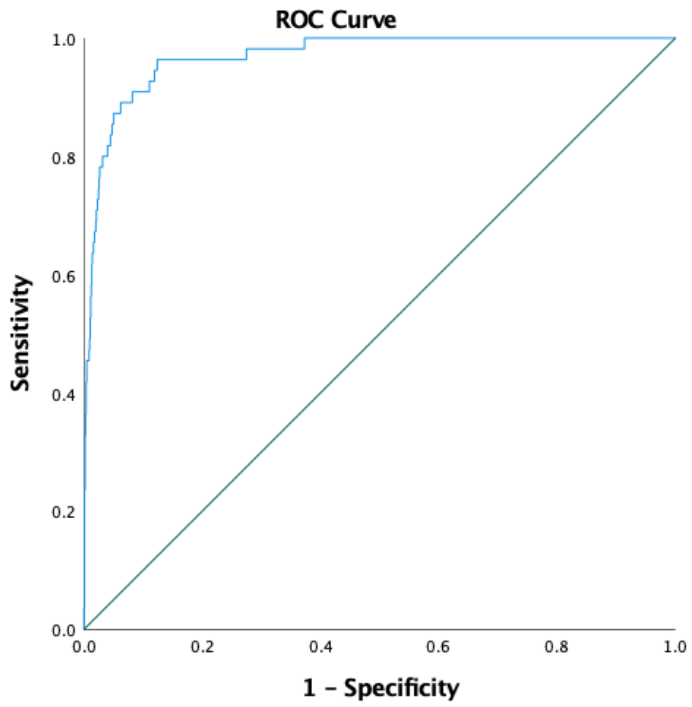

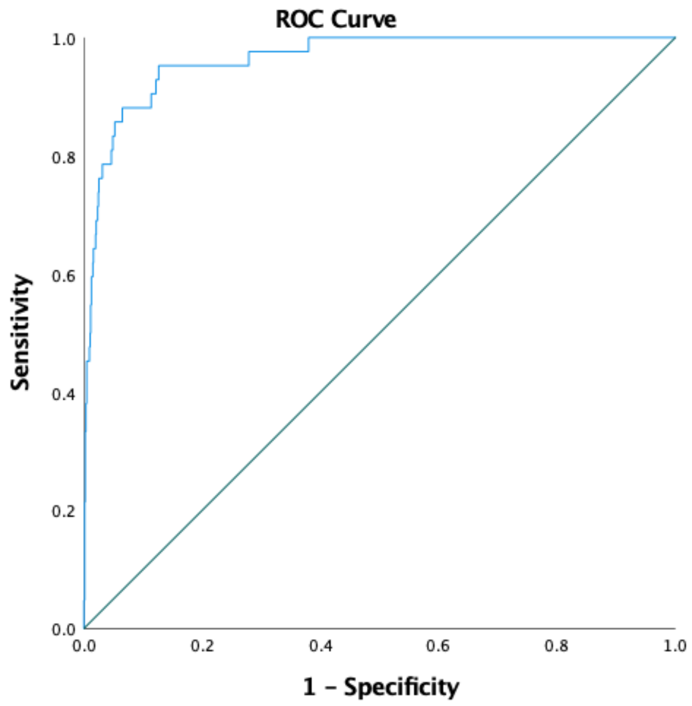

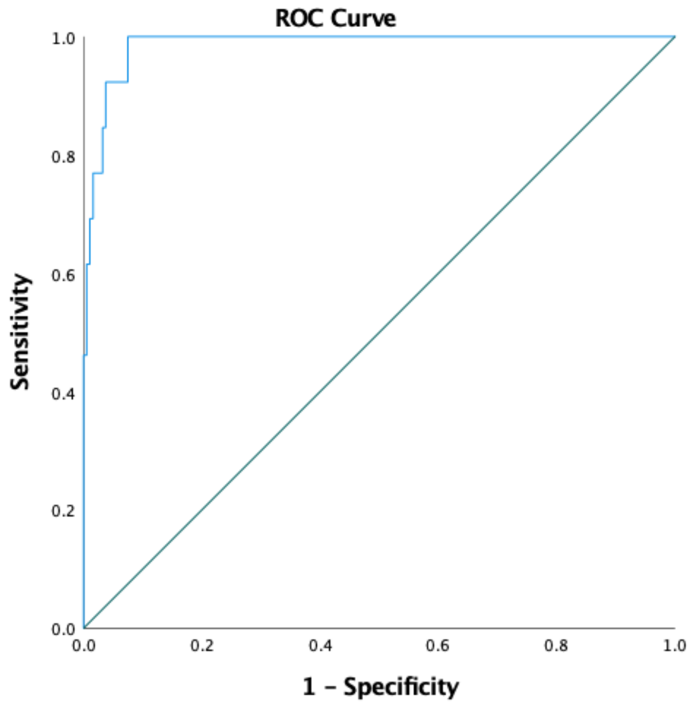
ROC Curve
Sensitivity
1 - Specificity
0.0
0.2
0.4
0.6
0.8
1.0

Supplementary Figure 4: ROC curves for measuring the performance of V-PLEX N in detecting COVID-19 infection with 6-months of preceding observation, overall (top), and among vaccinated (middle), and unvaccinated (bottom) cases

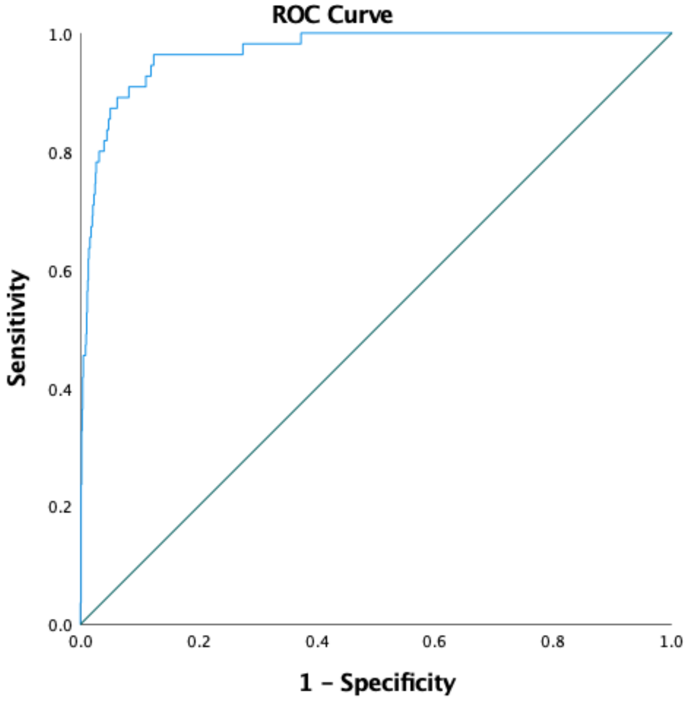

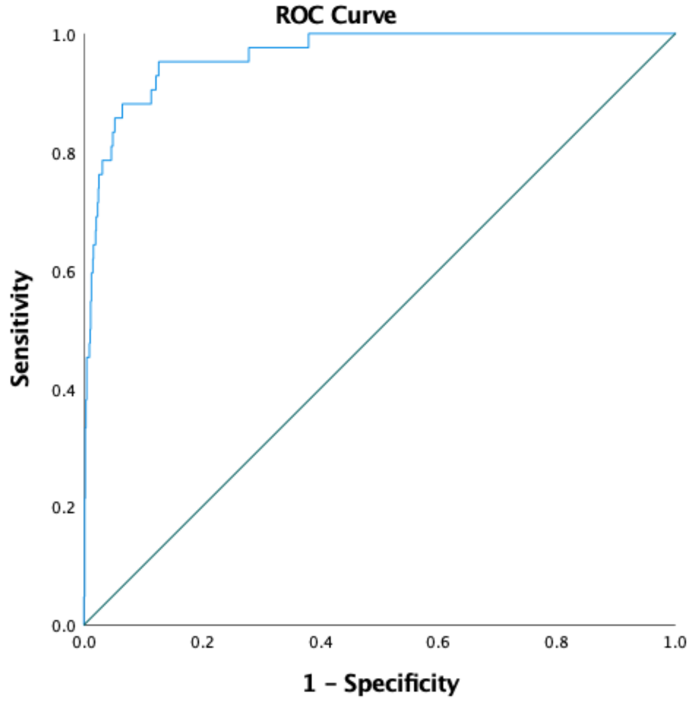

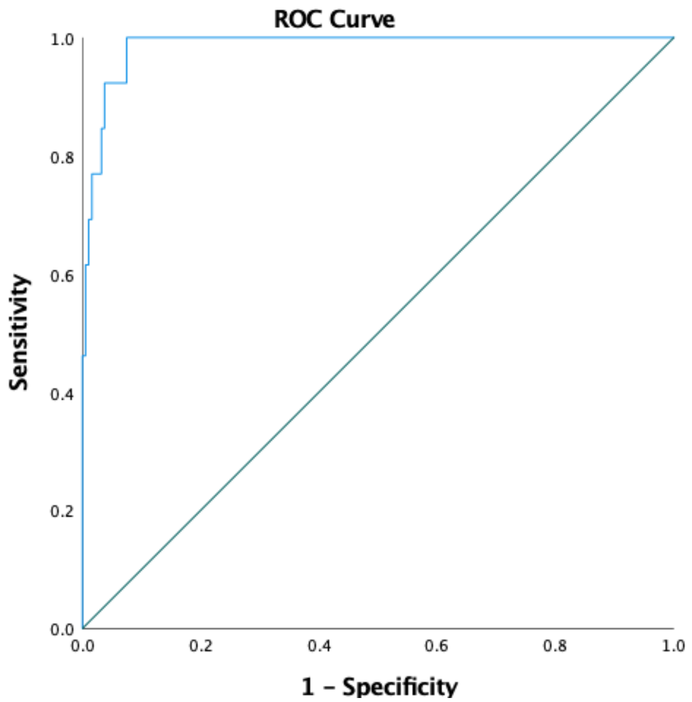
ROC Curve
1.0
0.8
0.6
0.4
0.2
0.0
Sensitivity
0.0
0.2
0.4
0.6
0.8
1.0
1 – Specificity

Supplementary Figure 5: ROC curves for measuring the performance of V-PLEX N in detecting COVID-19 infection with 3-months of preceding observation, overall (top), and among vaccinated (middle), and unvaccinated (bottom) cases

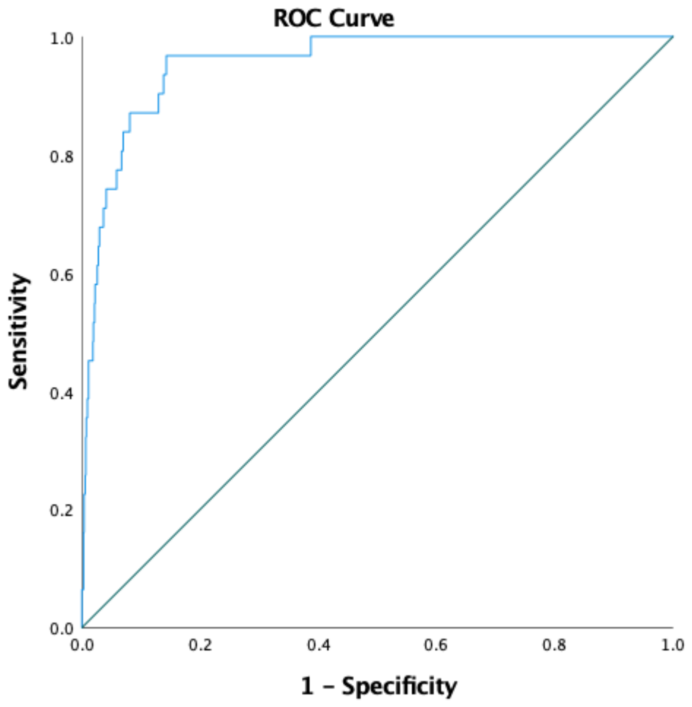

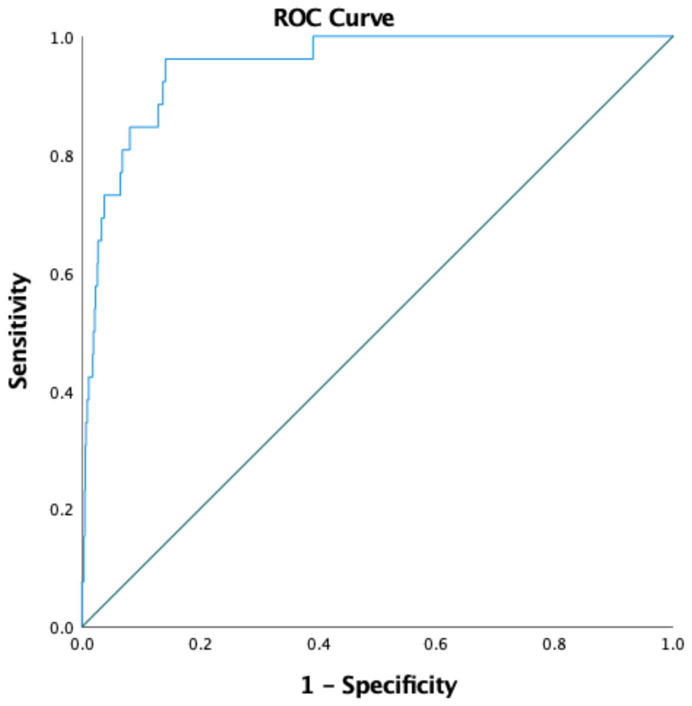

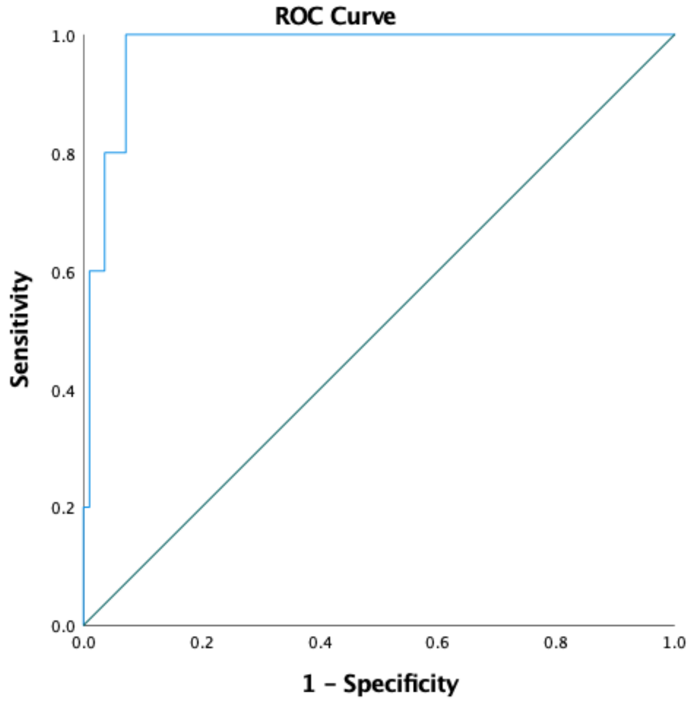

ROC Curve
Sensitivity
1 - Specificity
0.0
0.2
0.4
0.6
0.8
1.0
